# Training is not enough: child and adolescent psychiatry clinicians’ impressions of telepsychiatry during the first COVID-19 related lockdown

**DOI:** 10.1007/s00787-022-02042-2

**Published:** 2022-08-20

**Authors:** Vlad-Alexandru Rusu, R. M. van der Lans, R. R. J. M. Vermeiren, K. Hauber, J. M. de Lijster, R. J. L. Lindauer, A. Nugter, P. J. Hoekstra, L. A. Nooteboom

**Affiliations:** 1 Curium LUMC, Oegstgeest, The Netherlands; 2 Karakter, Wageningen, The Netherlands; 3 de Bascule, Duivendrecht, The Netherlands; 4 GGZ Noord-Holland-Noord, Heerhugowaard, The Netherlands; 5 Accare, Assen, The Netherlands

**Keywords:** Telepsychiatry, COVID-19, Child and Adolescent, Telemedicine, Psychiatry

## Abstract

To ensure the continuity of care during the COVID-19 pandemic, clinicians in Child and Adolescent Psychiatry (CAP) were forced to immediately adapt in-person treatment into remote treatment. This study aimed to examine the effects of pre-COVID-19 training in- and use of telepsychiatry on CAP clinicians’ impressions of telepsychiatry during the first two weeks of the Dutch COVID-19 related lockdown, providing a first insight into the preparations necessary for the implementation and provision of telepsychiatry during emergency situations. All clinicians employed by five specialized CAP centres across the Netherlands were invited to fill in a questionnaire that was specifically developed to study CAP clinicians’ impressions of telepsychiatry during the COVID-19 pandemic. A total of 1065 clinicians gave informed consent and participated in the study. A significant association was found between pre-COVID-19 training and/or use of telepsychiatry and CAP clinicians’ impressions of telepsychiatry. By far, the most favourable impressions were reported by participants that were both trained and made use of telepsychiatry before the pandemic. Participants with either training or use separately reported only slightly more favourable impressions than participants without any previous training or use. The expertise required to provide telepsychiatry is not one-and-the-same as the expertise that is honed through face-to-face consultation. The findings of this study strongly suggest that, separately, both training and (clinical) practice fail to sufficiently support CAP clinicians in the implementation and provision of telepsychiatry. It is therefore recommended that training and (clinical) practice are provided in conjunction.

## Introduction

The COVID-19 pandemic has led to unprecedented disruptions in health care worldwide. Among the affected fields was Child- and Adolescent Psychiatry (CAP). Measures and restrictions employed to exercise control over the spread of the virus left numerous clinicians in CAP unable to provide regular, day-to-day care in person. To ensure the continuity of care, clinicians in CAP were forced to immediately adapt in-person treatment to remote treatment. As a result, many children and youths abruptly had their former in-person treatment replaced by telepsychiatry (e.g. by phone, video calling, or eHealth modules). Moreover, CAP clinicians were forced to quickly familiarize themselves with the required expertise, as they were generally not yet adept in the provision of telepsychiatry.

Previous studies have shown that telepsychiatry can be effectively employed to improve access to and availability of health care [1–6]. Furthermore, an increasing body of evidence suggests that in specific situations, the patient outcome as well as therapeutic alliance are at least equal to in-person treatment [7–9]. Telepsychiatry was even shown to be preferable in some cases, due to, for example, geographical restrictions, time constraints, or (developmental) disabilities [10, 11].

Before the lockdown, imposed by the Dutch government on the 15^th^ of March 2020, psychiatric care in the Netherlands was mainly offered in person. Similar to most countries, the implementation of telepsychiatry was hindered by clinical, technological, and administrative concerns of clinicians (e.g. safety, privacy, technological limitations, financial and regulatory issues, credentialing, and education) [1, 2, 12, 13], lack of investment [10], and unprepared care systems [12]. Additionally, given that in-person psychiatric care in the Netherlands was readily available at a distance relatively close to home, telepsychiatry was deemed unnecessary.

The compulsory, en masse transition to telepsychiatry presents a unique opportunity to study telepsychiatry in an uncontrolled and wholly realistic environment. Not only is telepsychiatry being implemented on a larger scale than ever before; in addition, the conditions offer a rare possibility to study telepsychiatry as an immediate response to emergency situations.

Studies in adult psychiatry show that telepsychiatry was well perceived among clinicians during the COVID-19 pandemic [14, 15]. However, we lack insight into the experiences of CAP clinicians. Moreover, although guidelines for the application of telepsychiatry in CAP exist [16], experiences from practice indicate that these guidelines were rarely considered during the first few weeks of COVID-19 restrictions in The Netherlands.

To provide insight into the preparations that are required for the implementation and provision of telepsychiatry during emergency situations and similar uncontrolled environments in CAP, this study aimed to examine the effects of pre-COVID-19 training and use of telepsychiatry on CAP clinicians’ impressions of telepsychiatry during the first two weeks of the Dutch COVID-19 lockdown.

## Methods

### Study participants

Data were collected in five specialised CAP centres in the western and northern provinces of the Netherlands. All clinicians (*n* = 3570) employed by the participating centres were considered eligible for inclusion. Of the 3570 clinicians approached, 1065 (29.8%) gave informed consent and participated in the study. An overview of the study participants’ demographic data is provided in Table 1. As expected, the vast majority of participants (91%) reported that they provided telepsychiatry in the past two weeks. In contrast, less than half (44%) reported that they were trained for the provision of telepsychiatry.

**Table 1:** Demographic data of participants (*n* = 1065)

Descriptives	*n*	%^a^
Clinician age		
≤ 30	230	21.6
31–40	364	34.2
41–50	246	23.1
51–60	164	15.4
≥ 60	61	5.7
Profession^b^		
Psychiatrist, Resident, Clinical-psychologist, Psychotherapist	155	14.6
Healthcare-psychologist, General remedial educationalist	239	22.4
Systemic therapist, Remedial educationalist, Psychologist	370	34.7
Social worker, District team worker, Child and family centre worker, Psychiatric nurse	300	28.2
Treatment offered^c^		
Only support	116	10.9
Support and supportive treatment	105	9.9
Support and change-oriented treatment	188	17.7
Support, supportive treatment and change-oriented treatment	479	45.0
Only supportive treatment	30	2.8
Supportive treatment and change-oriented treatment	50	4.7
Only change-oriented treatment	93	8.7
Client age		
Only < 12	75	7.0
< 12 and ≥ 12	108	10.1
< 12 and parents	55	5.2
< 12, ≥ 12 and parents	271	25.4
Only ≥ 12	324	30.4
≥ 12 and parents	161	15.1
Only parents	63	5.9

^a^If rows do not add up to 1065, this is due to missing values

^b^For this study, professions were grouped into four broad categories. Categories were defined based on the required level of education; work activities; and the “BIG−register”, a recordkeeping establishment for healthcare professionals in the Netherlands. From top to bottom, the groups are arranged from highest requirements to lowest. The professions are categorized according to the Dutch model of mental healthcare

^c^Type of treatment offered was grouped into seven categories based on the type and scope of treatment

### Measures

A web-based, self-report questionnaire* was specifically developed by the authors to study CAP clinicians’ impressions of telepsychiatry during the COVID-19 pandemic (Appendix A). The content of the questionnaire was designed based on current literature on the use of telepsychiatry [1, 9–13], and inquired about the use of telepsychiatry, training in the use of telepsychiatry, clinicians’ impressions of telepsychiatry, and clinicians’ age, profession, types of treatment offered, and client age categories. Clinicians’ impressions of telepsychiatry were measured through a self-assessment on a scale of 1 to 10 and five scaled statements, meticulously covering three topics: (1) clinicians’ perceptions of the effectiveness of telepsychiatry compared to face-to-face treatment, (2) clinicians’ perceptions on the impact of telepsychiatry on the dyadic therapeutic alliance, and (3) clinicians’ perceptions on the appropriateness of available technical means.

### Design and procedures

Data collection commenced on April 1st, 2020, two weeks after the first COVID-19-related lockdown in the Netherlands. The questionnaire was distributed among all eligible clinicians through CASTOR, an online platform for capturing and integrating data. Captured data were converted to an SPSS dataset for further analyses. Four mutually exclusive groups were formed based on CAP clinicians’ pre-COVID-19 training in- and/or use of telepsychiatry (no/no (*N* = 749 (70.3%)), yes/no (*N* = 81 (7.6%)), no/yes (*N* = 116 (10.9%)), yes/yes (*N* = 119 (11.2%))).

Data collection was approved by the Leiden University Medical Centre Ethics Committee, determining that the study did not require to be subject to evaluation based on the Medical Research Involving Human Subject Act (03-04-2020, N20.058). Safe from the time requirement of five minutes per measurement, participating in the study was not expected to be detrimental to clinicians. Participation was voluntary and participants and participating centres received no compensation.

### Missing data

Missing responses were imputed through Multiple Imputation (MI): a way of dealing with nonresponse bias by estimating multiple probable values for every missing response through a prediction model. MI was performed using IBM SPSS statistics version 25. Before imputation, 11.2% of item responses were missing, with 26.7% of cases counting one or more missing values. As recommended by van Buren and colleagues, 40 datasets were imputed [17]. This resulted in 40 datasets for analysis.

All analyses were performed as Multivariate Analyses of (Co)Variance (MAN(C)OVA). The study, therefore, reports test-values and p-values in ranges (i.e. lowest and highest values), accompanied by the number of significant p-values across the 40 datasets. This practice sticks closest to the principal argument behind MI, namely that we cannot ‘know’ what value to impute, and that it is good practice to inform readers about the uncertainty of the imputations [17, 18]. However, to avoid uncertainty in the interpretation of the significance of the results, null hypotheses were only rejected when all *p* values, across all 40 datasets, were significant. The alpha criterion was set at 0.05.

### Statistical analysis

CAP clinicians’ impressions of telepsychiatry were reported as mean with standard deviation (Table 2). The number of CAP clinicians that reported training in the use of telepsychiatry before the lockdown was compared to the number of CAP clinicians that reported training in the use of telepsychiatry during the lockdown. The same principle was applied to the reported use of telepsychiatry.

**Table 2:** Overall impressions of telepsychiatry: scaled statements and self-assessment

Statements^a,b^ and self-assessment^b^	Mean	SD
Telepsychiatry is just as effective as face-to-face treatment	4.74	1.77
My competence in telepsychiatry is comparable to my competence in face-to-face treatment	4.42	1.90
I have sufficient technical resources at my disposal to apply telepsychiatry	5.74	2.15
Therapeutic alliance cannot be established through telepsychiatry alone	5.26	2.08
Possibilities to maintain therapeutic alliances in telepsychiatry are comparable to possibilities in face-to-face treatment	5.59	1.87
Based on the telepsychiatry you have provided in the past two weeks, what grade would you give yourself as a practitioner on average?	6.55	1.08

^a^Agree/disagree on a scale of 1–10. 1 = completely disagree. 10 = completely agree

^b^Translated from Dutch to English

Preliminary MANOVA tests were used to identify potential covariates. Associations between CAP clinicians’ impressions of telepsychiatry and their (1) age, (2) profession, (3) centre of employment, (4) type of treatment offered, and (5) client age groups, were tested. To correct for the identified covariates, a MANCOVA-test was used to analyse the effects of pre-COVID-19 training in- and/or use of telepsychiatry on CAP clinicians’ impressions of telepsychiatry. The individual effects of the four possible combinations of pre-COVID-19 training and/or use of telepsychiatry on CAP clinicians’ impressions of telepsychiatry were analysed using Estimated Marginal Means (EMM). All statistical analyses were performed with IBM SPSS statistics version 25.

## Results

### Training in- and use of telepsychiatry

Clinicians’ reports showed a drastic increase in both training in- and use of telepsychiatry after the lockdown came into force. The number of clinicians that were trained in the use of telepsychiatry increased from 202 (19%) to 469 (44%), and the number of clinicians that made use of telepsychiatry increased from 234 (22%) to 969 (91%).

### Impressions of telepsychiatry

Clinicians’ overall impressions of telepsychiatry during the first two weeks of the lockdown were modest, scoring between 4.4 and 6.6 on a scale of 1–10. A detailed overview of CAP clinicians’ overall impressions of telepsychiatry is provided in Table 2.

### Covariates

The following covariates were found to have a significant association with CAP clinicians’ overall impressions of telepsychiatry: *age* (*F* (24.0, 3650.3) = 2.2–3.0, *p* =  < 0.001–0.001, < 0.05 in 40/40 datasets; *Wilk's Λ* = 0.93–0.95); *centre of employment*, (*F* (24.0, 3560.3) = 2.0–3.0, *p* = 0.000–0.002, < 0.05 in 40/40 datasets; *Wilk's Λ* = 0.93–0.96); and *profession* (*F* (18.0, 2959.0) = 2.8–5.2, *p* = 0.000 in 40/40 datasets; *Wilk's Λ* = 0.92–0.95)*.* Not significantly associated were: *type of treatment offered*, (*F* (36.0, 4579.7) = 1.1–2.2, *p* =  < 0.001–0.309, < 0.05 in 24/40 datasets; *Wilk's Λ* = 0.93–0.96); and *client age groups*, *F* (42.0, 4895.6) = 0.7–1.6, *p* = 0.008–0.890, < 0.05 in 2/40 datasets; *Wilk's Λ* = 0.94–0.97).

### The effects of pre-COVID-19 training in- and/or use of telepsychiatry on clinicians’ impressions of telepsychiatry

Significant differences in CAP clinicians’ impressions of telepsychiatry were found between the four mutually exclusive groups of pre-COVID-19 training and/or use of telepsychiatry, *F* (18.0, 2933.6) = 2.2–3.1, *p* = 0.000–0.002, < 0.005 in 40/40 datasets; Wilk's Λ = 0.95–0.96.

**Fig. 1:**
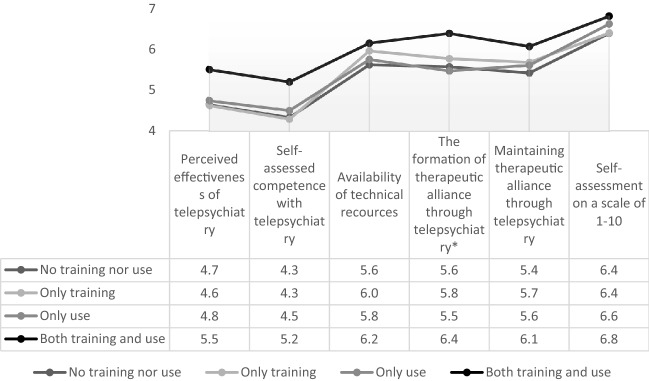
CAP clinicians’ impressions of telepsychiatry by group. *This statement and the corresponding values were reversed for the purpose of this figure, as the statement was initially formulated negatively, whereas a positive formulation allows for a better comparison with the other statements and self-assessment

CAP clinicians’ impressions of telepsychiatry by group are displayed in Fig. 1. By far, the most favourable impressions were reported by the “both training and use” group. Conversely, the least favourable impressions were reported by the “no training or use” group. Both the “only training” and “only use” groups reported only slightly more favourable impressions than the “no training or use” group.

## Lessons learned and consequences for the future

### Findings

This study aimed to examine the effects of pre-COVID-19 training and use of telepsychiatry on CAP clinicians’ impressions of telepsychiatry during the first two weeks of the Dutch COVID-19-related lockdown.

We found that, overall, CAP clinicians’ impressions of telepsychiatry were modest. By far, the most favourable impressions were reported by participants who had not only received training but also made use of telepsychiatry before the pandemic. Conversely, the least favourable impressions were reported by participants without previous training or use. Participants with either training or use separately reported only slightly more favourable impressions than participants with neither.

Our findings contradict previous literature, published during the COVID-19 pandemic, reporting highly favourable attitudes toward telepsychiatry and the benefits of bolstering training [14, 15]. Although methodologically similar; relying on a questionnaire querying clinicians’ attitudes towards telepsychiatry across multiple mental health centres; there are three primary differences between previous studies and ours. The first, and perhaps most evident difference lies in the studies’ setting. While the previous studies were conducted in adult psychiatry, our study was conducted in CAP. Not only are clients in CAP vastly different from clients in adult psychiatry, the client’s parents are typically involved as well; adding a layer of complexity to telepsychiatry consultation. The differences in client characteristics and the number of individuals involved may well attribute to why our findings contradict previous literature. The second difference lies in the content of the questionnaires. As all questionnaires were developed by the authors themselves, the questionnaires were not standardized between studies. This may hinder the comparability of the studies’ results. Third, the previous studies were conducted in the USA, whereas our study was conducted in the Netherlands. Cultural or national differences may have affected clinicians’ attitudes towards telepsychiatry.

### Implications

The pandemic has highlighted the employability of telepsychiatry as a response to emergency situations. Although it is admirable that many clinicians have been able to quickly (and without proper training or practice) adapt in-person treatment to remote treatment, the results of this study strongly suggest that telepsychiatry falls short of expectations if clinicians are not properly prepared.

Our findings suggest that, separately, both training and (clinical) practice fail to sufficiently support CAP clinicians in the implementation and provision of telepsychiatry. It is, therefore, recommended that training and (clinical) practice are provided in conjunction. The necessity of both training and (clinical) practice for CAP clinicians that are already experienced in (face-to-face) psychiatry, suggests that the expertise that is required to provide telepsychiatry is not one-and-the-same as the expertise that is honed through face-to-face consultation. This supports the findings of Crawford et al., affirming that telepsychiatry requires explicit competency development [19].

In response to the lack of a standard in telepsychiatry training [20–22], a competency-based education framework was proposed by Hilty et al. [22]. The competencies within this education framework were based on the ACGME, CanMEDS and andragogy, and were split by milestone levels via the Dreyfus model (novice, advanced beginner, competent, proficient, expert). Examples of defined competencies include ‘engagement and interpersonal skills’, ‘Safety’ and ‘Technology operation’. This educational framework provides a solid foundation for telepsychiatry training.

### Limitations

The strength of this study lies in the fact that it was set up quickly yet thoroughly. The study, therefore, offers unique insights into CAP clinicians’ first experiences with telepsychiatry during the COVID-19 pandemic; however, the study was not without limitations.

First, all clinicians employed by the participating institutes were approached. This may have resulted in a seemingly lower response rate, as clinicians that were not directly working with patients may have received the questionnaire. Second, due to the scarcity of literature on telepsychiatry as a response to emergency situations, the questionnaire was conceptualized based on core elements of regular telepsychiatry. Third, three of the statements that were used to assess CAP clinicians’ impressions of telepsychiatry were formulated in a way that compares telepsychiatry to face-to-face psychiatry. Although it was implied that a higher score means a more positive impression of telepsychiatry, these questions did not provide unequivocal instructions. Fourth, no distinction was made between types of training or the duration of training.

### Recommendations for future research

As telepsychiatry has proven to be a vital asset in ensuring the continuity of care during emergency situations, more studies are necessary to supplement the very limited knowledge on both the implementation and provision of telepsychiatry as a response to emergency situations. The knowledge gaps are vast and the opportunities for future research are abundant.

For example, it is unknown whether literature on telepsychiatry under normal circumstances also applies to telepsychiatry as a response to emergency situations. Moreover, although our study strongly suggests that simply adapting in-person treatment to remote treatment is highly ineffective if not properly prepared, and both training and (clinical) practice are necessary, this is hardly a detailed guideline. To this end, we hope that our study may serve as the starting point for future research, and we kindly encourage research that expands on our findings and fleshes out the necessary details.

## Data Availability

Not applicable.

## Appendix A: Questionnaire topics

**Table:**

Topic	Type of question(s)
Age	Multiple choice
Profession	Multiple choice (multiple answers possible)
Types of treatment offered	Multiple choice (multiple answers possible)
Age of clients	Multiple choice (multiple answers possible)
Use of telepsychiatry	Multiple choice
Training in the use of telepsychiatry	Multiple choice
Telepsychiatry modalities employed	Multiple choice (multiple answers possible)
Hours spent using telepsychiatry	Scale
Self rating	Scale
Perceived effectiveness of telepsychiatry	Scaled statement
Perceived competence with telepsychiatry	Scaled statement
Technical resources	Scaled statement
Instructions provided for the use of telepsychiatry	Scaled statement
Perceived suitability of telepsychiatry for crisis sensitive clients	Scaled statement
Forming therapeutic relationships through telepsychiatry	Scaled statement
Maintaining therapeutic relationships through telepsychiatry	Scaled statement
Experiences with tension increasing interventions through telepsychiatry	Scaled statement
Perceived motivation of clients with telepsychiatry	Scaled statement
Perceived client satisfaction with telepsychiatry	Scaled statement
Future use of telepsychiatry	Scaled statement
Open text field for additional comments	Open text field
